# Perception of Cardio-Kidney-Metabolic Risk and Health Literacy in Women: Quantitative and Qualitative Insights into Lifestyle and Prevention

**DOI:** 10.3390/jcm15207902

**Published:** 2026-10-13

**Authors:** Lovorka Bilajac, Mihaela Marinović Glavić, Andrej Belančić, Matea Bilobrk, Kristijan Zulle, Zlatko Trobonjača, Ana Jelaković, Bojan Jelaković

**Affiliations:** 1 Faculty of Medicine, University of Rijeka, 51000 Rijeka, Croatia; lovorka.bilajac@uniri.hr (L.B.); a.belancic93@gmail.com (A.B.); zlatko.trobonjaca@uniri.hr (Z.T.); anajelakovic9@gmail.com (A.J.); 2 Faculty of Health Studies, University of Rijeka, 51000 Rijeka, Croatia; kristijan.zulle@uniri.hr; 3 Teaching Institute of Public Health Primorje—Gorski Kotar County, 51000 Rijeka, Croatia; mateabilobrk5@gmail.com; 4 University Hospital Centre Zagreb, 10000 Zagreb, Croatia; jelakovicbojan@gmail.com; 5 School of Medicine, University of Zagreb, 10000 Zagreb, Croatia

**Keywords:** awareness, cardiometabolic risk factors, health literacy, mediterranean diet, metabolic syndrome, women’s health

## Abstract

**Background:** Women experience unique biological and life-course determinants of cardio-kidney-metabolic (CKM) health, yet the relationship between health literacy, CKM risk awareness, and preventive lifestyle behaviors remains poorly understood. We aimed to examine health literacy, CKM risk awareness, and Mediterranean diet adherence among women across different stages of adulthood and to qualitatively explore barriers and facilitators influencing the translation of health knowledge into preventive behaviors. **Methods:** We conducted a convergent mixed-methods study involving 198 women aged ≥18 years who completed a web-based questionnaire assessing health literacy, CKM risk awareness, Mediterranean diet adherence, and sociodemographic characteristics. Quantitative data were compared between women aged ≤50 years and >50 years using appropriate statistical tests. Two focus groups involving 14 women aged >50 years underwent inductive thematic analysis to explore perceptions of CKM health and preventive lifestyle behaviors. **Results:** Women older than 50 years had higher body mass index (27.79 ± 4.01 vs. 24.10 ± 4.20 kg/m^2^, *p* < 0.001), a greater prevalence of chronic disease (87.7% vs. 36.8%, *p* < 0.001), and higher Mediterranean Diet Scores (9.46 ± 4.54 vs. 7.99 ± 4.87, *p* = 0.035) than younger women, whereas health literacy (3.11 ± 0.48 vs. 3.07 ± 0.52, *p* = 0.599) and CKM risk awareness (26.25 ± 4.82 vs. 26.90 ± 5.33, *p* = 0.391) were comparable between age groups. Health literacy was moderately positively associated with CKM risk awareness (rho = 0.388, *p* < 0.001), whereas neither health literacy nor CKM risk awareness was significantly associated with Mediterranean diet adherence. Qualitative findings identified a persistent knowledge–behavior gap, with women demonstrating substantial experiential knowledge of chronic disease but reporting that work, caregiving responsibilities, and stress frequently impeded implementation of healthy lifestyle behaviors. **Conclusions**: Although health literacy was positively associated with CKM risk awareness, neither was significantly associated with Mediterranean diet adherence. These findings highlight a gap between health-related knowledge and dietary practice and support preventive strategies that combine health education with behavioral and environmental support.

## 1. Introduction

Cardiometabolic risk comprises a group of interconnected factors such as hypertension, dyslipidemia, insulin resistance, type 2 diabetes, obesity, and physical inactivity. In women, this issue requires particular attention because risk is influenced not only by general lifestyle habits, but also by sex-specific life stages and experiences, including pregnancy, gestational diabetes, menopause, and hormonal changes. Although cardiovascular disease has historically been perceived as a predominantly male health problem, previous studies indicate that women may underestimate their own risk, even when established risk factors are present [1,2]. Risk perception plays an important role in prevention, as it may influence whether individuals recognize the need for lifestyle change, seek medical advice, or participate in preventive programs. This is especially relevant for women with a history of adverse pregnancy outcomes, such as gestational diabetes or hypertensive disorders of pregnancy, which are established markers of increased future cardiovascular and metabolic risk. However, many women do not recognize these complications as indicators of their long-term health risk, suggesting a mismatch between objective risk and perceived vulnerability [3]. Limited awareness of the association between pregnancy-related complications and future cardiovascular risk suggests that objectively increased medical risk is not always accompanied by an appropriate perception of personal vulnerability. In this context, health literacy may play an important role as it encompasses the ability to access, understand, evaluate, and apply health information needed to make informed decisions. Higher levels of health literacy are associated with better recognition of cardiovascular risk factors, better communication with health care providers, and greater engagement in preventive health behaviors [4,5]. However, knowledge of cardiovascular risk among women remains inadequate and limited recognition of the long-term cardiovascular consequences of pregnancy-related cardiometabolic complications suggests gaps in risk communication and health literacy [2,6].

Despite increasing recognition of the importance of cardiovascular risk prevention in women, important gaps remain in understanding how women perceive their own cardiometabolic risk and how this perception relates to health-related behaviors. Previous studies have shown that women may have limited knowledge of cardiovascular risk factors and may underestimate their personal risk, while health literacy has been associated with better recognition of cardiovascular risk factors and greater engagement in preventive behaviors [2,4,5]. However, awareness and knowledge do not necessarily result in sustained lifestyle change. Health literacy may influence health behavior through several pathways, but its relationship with actual preventive practices is also shaped by individual, social, and contextual barriers [7,8,9]. In addition, women’s cardiometabolic risk is influenced by different life-course experiences, including reproductive and menopausal transitions, suggesting that risk perception and preventive needs may vary across adulthood [3,6]. A further gap concerns the relationship between cardiometabolic risk awareness, health literacy, and specific lifestyle behaviors, particularly dietary practices, among women at different stages of adulthood. While previous research has addressed cardiovascular risk knowledge, health literacy, and barriers to prevention, these aspects have often been examined separately [2,4,5,8,9]. Less is known about whether greater awareness of cardiometabolic risk is accompanied by healthier dietary practices and, importantly, what factors may explain a potential discrepancy between knowledge and everyday practice. Addressing this gap is relevant for prevention because identifying risk awareness alone may be insufficient if women face practical, social, or emotional barriers that limit their ability to translate health knowledge into sustained preventive behaviors. Therefore, the present mixed-methods study was designed to assess health literacy, cardio-kidney-metabolic risk awareness, and Mediterranean diet adherence among women across different stages of adulthood, to examine differences according to age, and to qualitatively explore the experiences, barriers, and facilitators that may influence the translation of health knowledge into preventive lifestyle behaviors.

## 2. Materials and Methods

### 2.1. Study Design

A mixed-methods design, combining quantitative and qualitative approaches, was used to obtain a more comprehensive insight into women’s perceptions of cardiometabolic risk and their levels of health literacy. The study was conducted between March and June 2026. The quantitative component enabled the systematic assessment of sociodemographic characteristics, health literacy, and knowledge of cardiometabolic health and related risk factors, while the qualitative component provided a deeper understanding of participants’ experiences, attitudes, and perceptions related to cardiometabolic risk and prevention. Quantitative and qualitative data were analyzed separately and integrated during interpretation using a convergent mixed-methods approach. The inclusion criteria were being female, being 18 years of age or older, being able to understand the Croatian language, and providing electronic informed consent before completing the questionnaire. Women were eligible regardless of their health status or the presence of chronic disease. This approach was intentional, as the study aimed to examine health literacy, cardiometabolic risk awareness, and preventive lifestyle behaviors across women with different health experiences and stages of adulthood, reflecting the broader population to which preventive strategies may be applied. At the same time, the presence of chronic disease was considered an important characteristic when interpreting differences in cardiometabolic risk awareness and health-related behaviors.

### 2.2. Quantitative Component: Structured Questionnaire

Data were collected using an anonymous self-administered web-based questionnaire developed in Google Forms and accessible via computers, tablets, and smartphones. Participants were recruited using a non-probability convenience sampling approach. The research team distributed the questionnaire through electronic communication channels, including email, allowing women from different geographic locations to participate. Participation was voluntary, and the questionnaire was accessible to women aged ≥18 years who understood Croatian and provided electronic informed consent. Incomplete questionnaires were excluded, and all eligible completed responses were included in the final analysis (*n* = 198). As the survey was anonymous, duplicate responses could not be formally identified. In addition, the completion rate could not be calculated because information on the number of individuals who accessed or initiated the questionnaire was not available. An a priori sample-size calculation was not performed, and all eligible participants who completed the questionnaire during the study period were included. Height and body weight were self-reported, and body mass index (BMI) was calculated from the reported values. Chronic non-communicable diseases were also self-reported based on whether a physician had ever diagnosed the participant with one or more of the following conditions: coronary heart disease or myocardial infarction, hypertension, high blood cholesterol, stroke or cerebrovascular disease, diabetes mellitus, chronic lung disease (including chronic bronchitis, emphysema, or chronic obstructive pulmonary disease - COPD), asthma, arthritis (including osteoarthritis or rheumatism), osteoporosis, cancer or malignant tumor, gastric or duodenal ulcer, Parkinson’s disease, cataract, hip fracture, or another chronic condition specified by the participant.

Before accessing the questionnaire, participants received information about the study purpose, voluntary participation, confidentiality, and their right to withdraw at any time, after which they provided electronic informed consent. The structured questionnaire collected sociodemographic data and assessed health literacy, adherence to the Mediterranean diet, and knowledge of cardiometabolic health and related risk factors. Marital status was included as a sociodemographic indicator, as partnership status may influence social support, health-related behaviors, and access to health information. Participants were categorized as single, married/cohabiting, divorced, or widowed. Education level was assessed as a sociodemographic characteristic and categorized according to the highest level of completed education. Education was considered an important determinant of health literacy, health information processing, and health-related behaviors. Monthly household income was assessed as an indicator of socioeconomic status and categorized as ≤€1250 and >€1250, using a threshold approximately corresponding to the average monthly net salary in Croatia at the time of data collection.

Health literacy was assessed using the HLS-EU-Q16, a validated 16-item short form of the European Health Literacy Survey Questionnaire [10]. Each item was rated on a four-point response scale. In the present study, the health literacy score was calculated as the mean of the 16 item responses, resulting in a score ranging from 1 to 4, with higher scores indicating higher perceived health literacy. The original HLS-EU-Q16 was developed and validated by Sørensen et al. [10]. The HLS-EU-Q16 demonstrated excellent internal consistency in the present sample (Cronbach’s α = 0.91).

Cardio-kidney-metabolic (CKM) risk awareness was assessed using an eight-item questionnaire developed by the research team specifically for the present study, informed by existing literature on cardiometabolic risk awareness and the establish CKM risk awareness score. The questionnaire was developed and administered in Croatian and has not been previously validated. The items addressed the interconnected nature of cardiovascular, kidney, and metabolic health as well as awareness of major modifiable and non-modifiable risk factors and preventive measures. Each item was rated on a four-point Likert scale ranging from 1 (“not at all sure”) to 4 (“completely sure”). Item scores were summed to obtain a total CKM risk awareness score ranging from 8 to 32, with higher scores indicating greater CKM risk awareness. The scale demonstrated good internal consistency in the present sample (Cronbach’s α = 0.87).

Adherence to the Mediterranean dietary pattern was assessed using the Mediterranean Diet Serving Score (MDSS) questionnaire. Participants reported their usual frequency of consumption for each food group, and scores were assigned according to the MDSS recommendations, yielding a total score ranging from 0 to 24, with higher scores indicating greater adherence to the Mediterranean diet [11]. Based on the total score, participants were classified as having non-adherence (0–6), moderate adherence (7–14), or adherence (15–24) to the Mediterranean dietary pattern.

### 2.3. Qualitative Component: Focus Groups

The qualitative component consisted of two semi-structured focus groups involving six and eight participants, respectively. Participants were purposively selected from women aged >50 years who had completed the quantitative survey and expressed willingness to participate in the qualitative phase. This age group was intentionally selected to provide a more in-depth exploration of health perceptions and preventive behaviors during later adulthood, a life stage characterized by important cardiometabolic changes and a greater burden of chronic disease.

Both focus groups were moderated by L.B., a researcher with experience in public health and qualitative research and a member of the study team. The moderator had no clinical or supervisory relationship with the participants.

Thematic saturation was assessed iteratively during the analysis. After the second focus group, no substantially new codes or themes emerged, and the researchers considered that thematic saturation had been reached; therefore, no additional focus groups were conducted. The two focus groups were conducted on 25 and 26 May 2026, respectively, in a meeting room at the Faculty of Medicine, University of Rijeka, in a quiet setting that ensured privacy and facilitated open discussion. Each focus group lasted approximately 60 min. A semi-structured approach was used to encourage open discussion while ensuring that all predefined discussion domains were addressed. The 14 participants were women aged >50 years and represented diverse occupational backgrounds. No additional sociodemographic characteristics were collected as part of the qualitative component. The focus groups were conducted in Croatian. The quotations presented in the manuscript were translated into English by the authors, with attention to preserving their original meaning and context. To ensure anonymity, quotations were identified using participant codes indicating the focus group and participant number (FG1_P1, FG2_P1).

Participants were recruited using purposive sampling among women who completed the quantitative survey and expressed willingness to participate in the qualitative phase. A semi-structured focus group guide was developed to ensure consistency across discussions and included introductory questions, key thematic areas, and follow-up prompts addressing participants’ understanding of cardiometabolic risk, perceived barriers to prevention, lifestyle behaviors, and the role of health information in everyday decision-making. The full focus group guide is provided in the Supplementary Material (Supplementary File S1. Focus Group Discussion Guide). Before participation, all participants received information about the study and provided written informed consent. Focus group discussions were audio-recorded, transcribed verbatim, and analyzed using thematic analysis. The discussions were conducted in Croatian.

### 2.4. Ethical Considerations

The study was conducted in accordance with ethical principles for research involving human participants, including voluntary participation, informed consent, confidentiality, and the protection of participants’ personal data. Participants were informed about the purpose of the study, the nature of their participation, and their right to withdraw from the study at any time without consequences. Electronic informed consent was obtained before completion of the quantitative questionnaire, while written informed consent was obtained from participants before the focus group discussions.

Ethical approval for the study was obtained from the Ethics Committee for Biomedical Research of the Faculty of Medicine, University of Rijeka (approval date: 20 January 2026.; class number KLASA: 007-08/26-01/03; registry number URBROJ: 2170-1-42-04-3/1-26-4).

### 2.5. Statistical Analyses

Quantitative data collected through the structured questionnaire were entered into a Microsoft Excel database and checked for completeness and consistency prior to analysis. Continuous variables were presented as mean ± standard deviation (SD), while categorical variables were reported as frequencies and percentages. Analyses were performed using available-case data; no imputation of missing values was performed. The number of observations was reported where data were missing. Data management was performed using Microsoft Excel, and statistical analyses were conducted using Statistica version 14 (TIBCO Software Inc., Palo Alto, CA, USA).

For the quantitative analysis, participants were categorized into two age groups (≤50 years and >50 years). The age threshold of 50 years was selected as a pragmatic life-course cut-off reflecting the transition toward midlife and the menopausal period, which is relevant to changes in body composition and cardiometabolic risk in women [12,13]. This categorization was intended to facilitate comparison across different stages of adulthood and was not used as a proxy for menopausal status.

Comparisons between women in different life stages (≤50 years and >50 years) were conducted using independent-samples *t*-tests for continuous variables. Homogeneity of variance was assessed using Levene’s test. Although some continuous variables showed departures from normality, independent-samples *t*-tests were retained given the sample sizes and absence of evidence of unequal variances. Sensitivity analyses using the Mann–Whitney U test yielded the same conclusions regarding statistical significance. Categorical variables were compared using Pearson’s chi-square (χ^2^) test; exact tests were used when expected cell counts were small. Effect sizes for continuous between-group comparisons were expressed as Cohen’s d, and mean differences are reported with 95% confidence intervals. Associations between Mediterranean Diet Score, health literacy score, and CKM risk awareness score were assessed using Spearman’s rank correlation coefficient (rho). Correlation coefficients were interpreted as weak (<0.30), moderate (0.30–0.49), or strong (≥0.50). Statistical significance was set at *p* < 0.05, and all tests were two-sided.

Focus group discussions were audio-recorded, transcribed verbatim, and analyzed using inductive thematic analysis. The discussion guide provided predefined topic domains to structure the conversations, but these domains were not treated as analytical themes. Two researchers independently reviewed the transcripts, performed open coding, and identified recurring patterns and concepts emerging from participants’ responses. Codes were subsequently grouped into broader categories and themes through an iterative process. The final themes were therefore generated from the data rather than imposed a priori by the interview guide. Discrepancies were resolved through discussion until consensus was reached. Representative quotations were selected to illustrate key themes.

## 3. Results

### 3.1. Quantitative Results

A total of 198 women participated in the study, including 68 women aged ≤50 years and 130 women aged >50 years. Significant differences were observed in sociodemographic characteristics between age groups. Older women were more frequently married or widowed, whereas younger participants were predominantly single (*p* < 0.001). Significant differences were also observed in household composition and household income distribution (both *p* < 0.001). Educational level differed between groups (*p* = 0.044). The prevalence of at least one chronic non-communicable disease was substantially higher among women older than 50 years compared to younger women (87.7% vs. 36.8%, *p* < 0.001) (Table 1).

Women aged >50 years had significantly higher body mass index (BMI) values compared to younger participants (27.79 vs. 24.10 kg/m^2^, *p* < 0.001). In contrast, no significant differences were observed between age groups in health literacy scores (3.11 ± 0.48 vs. 3.07 ± 0.52, *p* = 0.599) or cardio-kidney-metabolic (CKM) risk awareness scores (26.25 ± 4.82 vs. 26.90 ± 5.33, *p* = 0.391). Mediterranean diet adherence was moderate in both groups; however, women aged >50 years had significantly higher Mediterranean Diet Scores (MDSS) than younger women (9.46 ± 4.54 vs. 7.99 ± 4.87, *p* = 0.035). Data are presented as mean ± SD. Mean differences were calculated as >50 years minus ≤50 years and are presented with 95% confidence intervals (CI). Cohen’s d is reported as the standardized effect size, p-values are based on independent-samples t-tests comparing women aged ≤50 years and >50 years (Table 2).

Spearman’s correlation analyses showed a moderate positive association between health literacy and CKM risk awareness (rho = 0.388, *p* < 0.001). In contrast, health literacy was not significantly associated with Mediterranean Diet Score (rho = 0.085, *p* = 0.234), and the association between CKM risk awareness and Mediterranean Diet Score was also not statistically significant (rho = 0.125, *p* = 0.081) (Table 3).

Exploratory analyses were conducted to examine associations between individual Mediterranean diet components and cardiometabolic risk awareness using Spearman’s rank correlation coefficient. These analyses were considered exploratory due to the multiple dietary components examined, and no adjustment for multiple comparisons was applied. In the exploratory analyses of individual dietary components, vegetable consumption showed a weak negative correlation with cardiometabolic risk awareness (rho = −0.155, *p* = 0.030). No significant associations were observed for fruit, fish, legumes, nuts, olive oil, meat, dairy products, sweets, or wine consumption.

### 3.2. Qualitative Results

Two focus groups involving a total of 14 community-dwelling women aged over 50 years were conducted. Participants represented diverse occupational backgrounds, including education, administration, healthcare, accounting, hospitality, trade, and industry. The discussions explored experiences related to health information-seeking, perceptions of cardiometabolic risk, lifestyle behaviors, and preventive health practices.

The inductive analysis identified three main themes: (1) health information-seeking, communication, and evaluation; (2) knowledge and perceptions of cardiometabolic risk; and (3) prevention and lifestyle habits. The findings indicate that the participants had practical knowledge about chronic diseases, but this knowledge was mostly shaped by personal experience, experiences of family members, and communication with healthcare professionals rather than through formal health education.

**Theme** **1.** *Health Information-Seeking, Communication, and Evaluation*.

Participants described varied experiences in seeking and evaluating health information. Some actively sought clarification from healthcare professionals, emphasizing the importance of obtaining sufficient explanations: *“I do not leave the doctor’s office until I have asked all my questions and everything is clear to me”* (FG2_P6). In contrast, others reported difficulties with communication and insufficient explanations: *“I ask questions, but the doctor never answers me properly”* (FG1_P3). When information received from healthcare professionals was perceived as insufficient, participants sought additional information from the internet, acquaintances working in healthcare, or their own experience. However, they expressed caution toward online health information and emphasized the importance of evaluating its credibility and source, as reflected in statements such as *“Who is the author?” and “I do not trust the Internet”* (FG2_P6).

**Theme** **2.** *Knowledge and Perceptions of Cardiometabolic Risk*.

Participants did not spontaneously use the formal term cardio-kidney-metabolic syndrome during the discussions. Nevertheless, they demonstrated substantial awareness of cardiovascular disease, diabetes, and kidney disease. Their understanding was primarily based on personal and family experiences and was frequently expressed through disease consequences rather than medical definitions. For cardiovascular disease, participants frequently associated the condition with mortality: *“My whole family died from heart disease”* (FG2_P5).

Kidney disease was commonly associated with dialysis and kidney stones. Diabetes was often linked to severe complications: *“Leg amputation” and “Complications in life”* (FG2_P6, FG1_P6).

Although participants did not spontaneously use the formal CKM concept, many intuitively perceived a connection between cardiovascular, renal, and metabolic disorders, describing the human body as an interconnected system:

*“The whole body is connected”* (FG2_P1), *“Everything is connected through the blood vessels and the heart”* (FG1_P5).

**Theme** **3.** *Prevention and Lifestyle Habits*.

Participants generally believed that chronic diseases can be prevented through personal responsibility and self-discipline. Healthy eating, physical activity, adequate sleep, and stress management were consistently identified as important preventive measures.

However, most participants reported that they had paid little attention to these lifestyle factors during their working years. Occupational responsibilities, caregiving duties, and lack of time were recurrently described as barriers to engaging in preventive health behaviors. Preventive awareness often emerged only after retirement or following the onset of chronic disease. At the same time, participants stated that during their working years they did not think enough about their own health because work and family responsibilities took priority. One participant summarized this period with the phrase: *“Work, work, work”* and *“We never thought about it”* (FG2_P5). Stress emerged as one of the most frequently cited determinants of health. Participants associated stress with deteriorating health and emphasized stress reduction, emotional well-being, and maintaining “peace of mind” as important components of disease prevention. This was reflected in statements such as *“Avoid stress”*, *“You should worry less”* (FG1_P5, FG2_P6), which captured the broader perception that stress management was an important component of prevention. Participants frequently described emotional well-being and effective stress management as essential components of healthy aging and disease prevention.

## 4. Discussion

The present mixed-methods study provides new insights into the relationship between health literacy, cardiometabolic risk perception, lifestyle behaviors, and dietary habits among women across different stages of adulthood. Although women older than 50 years had a higher mean BMI and a greater prevalence of self-reported chronic non-communicable diseases, they demonstrated levels of health literacy and cardiometabolic risk awareness comparable to those of younger women. At the same time, older women showed greater adherence to the Mediterranean diet. Health literacy was moderately associated with CKM risk awareness; however, neither health literacy nor CKM risk awareness was significantly associated with Mediterranean diet adherence. Qualitative findings further revealed that women possessed substantial experiential knowledge of chronic diseases, while their understanding was primarily shaped by personal and family experiences rather than formal CKM terminology. Taken together, these findings suggest that age-related differences in cardiometabolic health status may coexist with broadly similar levels of health literacy and CKM risk awareness, while the translation of knowledge into preventive behaviors may be influenced by broader biological, social, and life-course factors.

The higher BMI observed among women over 50 years is consistent with previous evidence demonstrating changes in body composition, including increased adiposity, during midlife and the menopausal transition [12,13,14]. However, BMI should be interpreted with caution in postmenopausal women, as it may not fully reflect adiposity and may underestimate obesity when compared with direct measures of body fat [15]. Although dietary quality appeared slightly more favorable in older women, age-related physiological changes may contribute to changes in body composition and weight regulation independently of dietary quality [13,14]. Because menopausal status was not directly assessed in the present study, these findings should not be interpreted as evidence of an independent effect of menopause on BMI. This finding suggests that improvements in dietary habits may not fully offset the metabolic changes occurring during midlife and later adulthood. Thus, the higher BMI observed in older women may reflect the combined influence of biological aging and lifestyle factors rather than poorer dietary quality alone.

Aging and midlife physiological changes may contribute to changes in body composition and weight regulation [13,15]. Consequently, body weight regulation during later adulthood depends on multiple interacting biological and behavioral factors rather than dietary quality alone [12,13]. Older women demonstrated greater adherence to the Mediterranean diet and reported more frequent consumption of several Mediterranean dietary components, including fruits, vegetables, legumes, fish, and olive oil. This pattern is consistent with evidence linking adherence to the Mediterranean diet with favorable cardiovascular outcomes [16].

The greater adherence observed in older women in our sample may reflect differences in dietary habits and health-related behaviors between age groups. Several explanations are possible. Older women may have more established meal routines, greater opportunities to prepare meals, or increased attention to diet in response to age-related health concerns. Generational differences in food choices may also contribute. These explanations should, however, be considered exploratory, as the present study did not directly assess the reasons underlying the observed differences in dietary habits. An important finding of the present study was the moderate positive association between health literacy and CKM risk awareness. This finding suggests that women with greater perceived ability to access, understand, evaluate, and apply health information also demonstrated greater awareness of CKM-related risk. However, neither health literacy nor CKM risk awareness was significantly associated with adherence to the Mediterranean diet. This suggests that health literacy may support recognition and understanding of health risks without necessarily leading to corresponding behavioral changes. These findings are consistent with evidence indicating that health literacy, although important, represents only one of multiple determinants of health behavior [17]. Behavioral change is influenced by additional factors, including motivation, self-efficacy, environmental opportunities, socioeconomic circumstances, competing priorities, and social support [8]. Consequently, health education alone may be insufficient to achieve sustained lifestyle change when practical and contextual barriers to behavior change are present.

The qualitative findings provide valuable context for interpreting the quantitative results. However, because the qualitative component included only women older than 50 years, these findings primarily reflect the experiences and perceptions of women in later adulthood and should not be generalized directly to younger women. Although participants demonstrated substantial awareness of chronic diseases, much of their knowledge originated from personal experience, family illness, or aging rather than structured health education. Women consistently recognized the importance of healthy eating, physical activity, stress management, and adequate sleep; however, many acknowledged that work responsibilities, caregiving roles, and lack of time had prevented them from adopting healthier lifestyles earlier in life. These qualitative findings provide context for the quantitative observation that, although health literacy was associated with CKM risk awareness, neither was significantly associated with Mediterranean diet adherence. Knowledge and awareness were present, but opportunities and circumstances often limited behavioral implementation. These findings suggest that health literacy and risk awareness alone may not be sufficient to promote preventive behavior when women lack time, opportunities, behavioral support, and enabling social conditions [18,19]. An additional finding emerging from the focus group discussions was the discrepancy between experiential knowledge and familiarity with the formal concept of cardio-kidney-metabolic syndrome. The formal CKM framework has only recently been introduced as an integrated approach to the interrelationships among metabolic risk factors, chronic kidney disease, and cardiovascular disease [20]. Participants did not spontaneously use the formal term cardio-kidney-metabolic syndrome during the discussion. However, they demonstrated substantial awareness of cardiovascular disease, diabetes, and kidney disease and recognized that these conditions were interconnected. Their understanding was primarily expressed through personal and family experiences and perceived disease consequences rather than through formal medical terminology. This finding is unsurprising given that the cardio-kidney-metabolic framework has only recently emerged in clinical practice and public health. Nevertheless, increasing public awareness of this integrated concept may facilitate earlier recognition of shared risk factors and encourage comprehensive preventive strategies rather than disease-specific approaches. Our findings suggest that health literacy and risk awareness alone may not be sufficient to translate knowledge into sustained dietary and preventive behaviors [17].

From a public health perspective, these findings suggest that preventive programs targeting women should extend beyond health education alone. Interventions should combine health literacy with practical behavior-change support, opportunities for physical activity, accessible healthy food choices, stress-management strategies, and supportive social environments. Particular attention should be given to midlife and other life-course transitions as opportunities to identify cardiometabolic risk factors and reinforce preventive behaviors. This includes systematic identification and management of established risk factors such as elevated blood pressure, in accordance with current hypertension recommendation [21]. Communication strategies should also use clear and accessible language to explain the interconnected nature of cardiovascular, renal, and metabolic health. Importantly, prevention should address the barriers identified in the qualitative findings, particularly limited time, work and caregiving responsibilities, and competing life priorities. Such an integrated approach may help bridge the observed knowledge–behavior gap and improve the translation of cardiometabolic risk awareness into sustainable preventive behaviors.

These findings are consistent with the growing emphasis on lifestyle medicine, which emphasizes that sustainable health behavior change requires not only knowledge but also appropriate behavioral strategies and ongoing support [22]. Bridging the knowledge–behavior gap should therefore become a central objective of future preventive programs aimed at improving women’s cardio-kidney-metabolic health across the life course.

### Strengths and Limitations

The principal strength of this study is its mixed-methods design, combining quantitative assessment of health literacy, cardio-kidney-metabolic risk awareness, and dietary behaviors with qualitative exploration of women’s experiences and barriers to prevention. This approach provided a deeper understanding of the knowledge–behavior gap and contributed to the emerging field of cardio-kidney-metabolic health by examining awareness across different stages of adulthood.

Several limitations should be considered. First, the cross-sectional design precludes conclusions about temporal or causal relationships, and reverse causation cannot be excluded. Second, self-reported dietary habits, chronic conditions, and health behaviors may be subject to recall and social desirability bias. Third, online recruitment may have introduced selection bias and limited representativeness. Several relevant determinants, including menopausal characteristics, adverse pregnancy outcomes, objectively measured physical activity, sleep, psychosocial factors, medication adherence, and healthcare utilization, were not assessed and may have influenced the findings. Socioeconomic status was assessed using broad indicators and may not fully capture relevant financial and environmental factors. The qualitative component involved a small, purposively selected sample from a single geographic setting; therefore, transferability may be limited, and focus group dynamics and social desirability may have influenced responses. Finally, the inclusion of women only and the absence of objective clinical biomarkers limit generalizability and subgroup analyses. Nevertheless, these limitations should be considered in relation to the study aim, which was to provide an in-depth understanding of health literacy and preventive behaviors rather than generate nationally representative estimates.

## 5. Conclusions

This mixed-methods study demonstrates that women across adulthood had broadly comparable levels of health literacy and CKM risk awareness, while adherence to the Mediterranean diet was higher among women over 50 years. Health literacy was positively associated with CKM risk awareness; however, neither health literacy nor CKM risk awareness was significantly associated with Mediterranean diet adherence. Older women also had a higher mean BMI and a higher prevalence of chronic non-communicable diseases. Qualitative findings highlighted that woman recognized the importance of healthy eating and other preventive practices, but reported barriers related to work, caregiving responsibilities, limited time, and competing life priorities. Together, these findings suggest that greater health literacy and risk awareness may not necessarily translate into preventive dietary practices. Future interventions should therefore combine health literacy with practical behavior-change support and enabling environments, particularly during midlife and other important life-course transitions, to support women’s long-term cardiovascular, kidney and metabolic health.

## Figures and Tables

**Table 1:** Sociodemographic Characteristics of Participants, *n* (%).

Characteristic	≤50 Years (*n* = 68)	>50 Years (*n* = 130)	*p*-Value
Age (average)	34.39	68.6	
Marital status			<0.001
Single	35 (51.5%)	20 (15.4%)	
Married/cohabiting	29 (42.6%)	71 (54.6%)	
Divorced	3 (4.4%)	1 (0.8%)	
Widowed	1 (1.5%)	38 (29.2%)	
Household composition			<0.001
Living alone	3 (4.4%)	46 (35.4%)	
Living with a partner, without children	8 (11.8%)	48 (36.9%)	
Living with a partner and children	25 (36.8%)	23 (17.7%)	
Living with children, without a partner	3 (4.4%)	7 (5.4%)	
Living in a shared household with other family members or relatives	29 (42.6%)	6 (4.6%)	
Education level			0.044
Secondary education	8 (11.7%)	31 (23.8%)	
Undergraduate degree	21 (30.9%)	42 (32.3%)	
Graduate degree	15 (22.1%)	32 (24.6%)	
University degree	24 (35.3%)	25 (19.2%)	
Monthly household income (€)			<0.001
≤1250	11 (16.2%)	71 (55.5%)	
>1250	57 (83.8%)	57 (44.5%)	
At least one chronic non-communicable disease	25 (36.8%)	114 (87.7%)	<0.001

Data are presented as *n* (%). Between-group comparisons were performed using Pearson’s chi-square test or an exact test where expected cell counts were small. Income data were missing for two women in the >50-year group; therefore, the denominator for income in this group was *n* = 128. Percentages may not sum to 100% because of rounding.

**Table 2:** Health Literacy, CKM Risk Awareness, and Mediterranean Diet Adherence According to Age Group (mean ± SD).

	≤50 Years (*n* = 68)	>50 Years (*n* = 130)	Mean Difference (95% CI)	Cohen’s d	*p*
BMI (kg/m^2^)	24.10 ± 4.20	27.79 ± 4.01	3.70 (2.49 to 4.90)	0.91	<0.001
Health Literacy score	3.07 ± 0.52	3.11 ± 0.48	0.04 (−0.11 to 0.18)	0.08	0.599
CKM awareness score	26.90 ± 5.33	26.25 ± 4.82	−0.64 (−2.12 to 0.83)	−0.13	0.391
Mediterranean Diet Score	7.99 ± 4.87	9.46 ± 4.54	1.48 (0.10 to 2.85)	0.32	0.035

**Table 3:** Associations between Health Literacy, CKM Risk Awareness, and Mediterranean Diet Adherence.

Association	*n*	Spearman’s Rho	*p*
Health literacy—CKM awareness	195	0.388	<0.001
Health literacy—MDSS	197	0.085	0.234
CKM awareness—MDSS	195	0.125	0.081

Associations were assessed using Spearman’s rank correlation coefficient.

## Data Availability

The original contributions presented in this study are included in the article. Further inquiries can be directed to the corresponding author.

## Supplementary Materials

The following supporting information can be downloaded at https://www.mdpi.com/article/10.3390/jcm15207902/s1, Supplementary File S1. Focus Group Discussion Guide.
