# Supplementary material for: Perception of Cardio-Kidney-Metabolic Risk and Health Literacy in Women: Quantitative and Qualitative Insights into Lifestyle and Prevention
Source: J Clin Med. 2026 Oct 13;15(20):7902. doi: 10.3390/jcm15207902 (PMC13705493; doi:10.3390/jcm15207902)
Supplement: Supplementary file 1 [file jcm-15-07902-s001.zip › jcm-4470719-supplementary.pdf]

Supplementary File S1. Focus Group Discussion Guide.
Role of Health Literacy in Reducing the Risk of Cardio-Renal-Metabolic Syndrome in Women

Developed for the study: KReMe50
Principal investigator: Lovorka Bilajac
Study team: Lovorka Bilajac, Mihaela Marinović Glavić
Funded by the University of Rijeka (Project No. uniri-iz-25-100)
Version 1.0, January 2026.

Number of focus groups: Two

**Topic**	The role of health literacy in reducing the risk of cardio-renal-metabolic syndrome in women
**Venue and time**	University of Rijeka, Faculty of Medicine, Brace Branchetta 20, Rijeka, Croatia; 25 and 26 May 2026
**Participants**	Women aged 50 years and older
**Duration**	45–60 minutes
**Focus group objective**	To explore attitudes, knowledge, and perceptions regarding health literacy and its importance in the prevention of cardio metabolic syndrome, including risk factors, preventive measures, and access to health information.

This document is an English translation of the original Croatian-language focus group discussion guide used in the study.

Moderator's Opening Script

Introduction for participants:
Welcome, and thank you for taking the time to participate in this focus group. Our discussion concerns the role of health literacy in reducing the risk of cardio-renal-metabolic syndrome. The aim is to explore how a better understanding of health can contribute to disease prevention and health management. As the moderator, my role is to guide the conversation and discussion. There are no right or wrong answers; what matters is that you openly share your experiences, opinions, and views. All information shared during the discussion will be treated confidentially, and individual responses will not be identifiable in any reports or publications.
To begin, please tell us your age, where you live, and your occupation.

Discussion Guide

Section	No.	Question / Prompt
**1. Opening questions**	1a	How would you describe your current state of health?
	1b	What are the most important or significant health problems for people in your age group?
	1c	Do you understand the test results, recommendations, or advice provided by your doctor?

	1d	Do you know what you should do and where you can find additional information? Do you feel comfortable asking your healthcare provider questions when something is unclear?
**2. Key discussion domains: Cardio-Renal-Metabolic Syndrome**	2a	When you hear terms such as high blood pressure, diabetes, high cholesterol, kidney disease, or cardiovascular disease, what is the first thing that comes to mind?
	2b	In your opinion, are all the conditions mentioned above connected?
	2c	When you think about your own health, what worries you most: your heart, blood pressure, blood sugar, cholesterol, kidneys, body weight, or something else?
**3. Health Literacy and Prevention**	3a	What contributes most to cardio-renal-metabolic syndrome, and do you think it can be prevented?
	3b	What does "preventing disease" mean to you?
	3c	Have you changed your lifestyle habits at any point in your life?
	3d	What can you do now to stay healthy, and what can the healthcare system do to support your health?
	3e	What type of education would be most useful to you: a conversation with a doctor, a workshop, written material, a video, a group meeting, individual counselling, or something else?
	3f	What advice would you give others about maintaining their health and preventing cardio-renal-metabolic syndrome?
	3g	Where do you usually obtain health-related information (e.g., healthcare professionals, internet, social media, family, friends)? How do you decide whether the information is reliable?

Conclusion

**Moderator task**	Summarise the main conclusions of the focus group.
**Closing**	Thank the participants for their time and contribution.

Focus group transcripts were analysed using inductive thematic analysis. Themes were derived from participants' responses through an iterative coding process rather than predefined categories.
